# Changes Coming to MDPI Journals: Digital Object Identifier (DOI) and Creative Commons Attribution License

**DOI:** 10.3390/molecules13051079

**Published:** 2008-05-02

**Authors:** Derek McPhee, Shu-Kun Lin

**Affiliations:** Molecular Diversity Preservation International (MDPI), Matthaeusstrasse 11, CH-4057 Basel, Switzerland; Fax: +41 61 302 8918; E-mails: mcphee@mdpi.org; lin@mdpi.org

Effective immediately authors and readers shall notice some minor yet significant changes to the papers published in this and other MDPI journals [1].

The first of these changes is the addition of Digital Object Identifier (DOI) numbers to each paper using the CrossRef system [2]. This was necessary to keep up with recent trends in the scientific publishing market, mainly to allow cross-referencing of citations and this action will ensure permanent retrievability of content through DOI-based links. The DOI numbers assigned to all articles published by MDPI will facilitate incoming links from other Publishers’ online content and MDPI will also strive cross-link its own citations to other Publishers’ online content over the next two years.

The second change is the addition of an Open Access logo above the individual journal title logos. Although we have been using this for some time now on the Tables of Contents of the various MDPI journals to indicate the level of accessibility of papers, we shall now also be adding them to the individual articles themselves to further emphasize our support for this publishing model (readers should note, however, that papers published in early volumes of MDPI journals may be Open Access even though this is not specifically indicated by a logo in the Tables of Contents).

Thirdly, we have been aware of the fact that the copyright line text previously used at the end of MDPI published papers was not in line with the Budapest-Bethesda-Berlin definitions on Open Access (commonly referred to as the BBB definitions) [3]. To remedy this we are currently moving to a Creative Commons (CC) "By Attribution" license v. 3.0 model. The MDPI journal *Marine Drugs* has already been published under such a license since January 2008. Other MDPI journals, including this one, will start publishing under this license in the current and June 2008 issues. All content previously published by MDPI will be released under the CC "By Attribution" license within a few months, as we roll out our new publication platform (now in testing) and the copyright line text has been changed to reflect this.

Finally, authors and readers will note a minor change to the names used for the various types of papers published in our journals. In order to align our naming scheme with the recommendations of the NLM [4], and after some deliberation among the production staff teams of the various MDPI journals, we have settled on the nomenclature *Communication*, *Article* (formerly *Full Paper* or *Full Research Paper*, depending on the journal) and *Review*. 
